# Editorial Expression of Concern: IRE1a constitutes a negative feedback loop with BMP2 and acts as a novel mediator in modulating osteogenic differentiation

**DOI:** 10.1038/s41419-018-1175-8

**Published:** 2018-11-09

**Authors:** F. J. Guo, R. Jiang, Z. Xiong, F. Xia, M. Li, L. Chen, C. J. Liu

**Affiliations:** 1 Department of Cell Biology and Genetics, Core Facility of Development Biology, Chongqing Medical University, Chongqing, China; 2 Laboratory of Stem Cells and Tissue Engineering, Chongqing Medical University, Chongqing, China; 3 Department of Rehabilitation Medicine, State Key Laboratory of Trauma, Burns and Combined Injury, Trauma Center, Institute of Surgery Research, Daping Hospital, Third Military Medical University, Chongqing, China; 4 Departments of Orthopaedic Surgery and Cell Biology, New York University School of Medicine, New York, NY USA; 5 Present Address: The Bashu School of Science, Chongqing, China

Addendum to: Cell Death and Disease (2014)5:e1239 10.1038/cddis.2014.194 published online 22 May 2014

The Editors-in-Chief are issuing an editorial expression of concern to alert readers that after publication of this article^1^ concerns have been raised with respect to the integrity of Figs. 1c, e, 2b, 6b, and 7a. Chongqing Medical University is investigating these concerns and appropriate editorial action will be taken once the outcome of this investigation is known. The authors do not agree with this notice.
